# Proinflammatory Cytokines (IL-1, -6, -8, -15, -17, -18, -23, TNF-α) Single Nucleotide Polymorphisms in Rheumatoid Arthritis—A Literature Review

**DOI:** 10.3390/ijms23042106

**Published:** 2022-02-14

**Authors:** Olga M. Koper-Lenkiewicz, Kinga Sutkowska, Natalia Wawrusiewicz-Kurylonek, Ewa Kowalewska, Joanna Matowicka-Karna

**Affiliations:** 1 Department of Clinical Laboratory Diagnostics, Medical University of Bialystok, Waszyngtona 15A, 15-269 Białystok, Poland; kinga.kijek1@gmail.com (K.S.); ewa.kowalewska12345@gmail.com (E.K.); matowic@umb.edu.pl (J.M.-K.); 2 Department of Clinical Genetics, Medical University of Bialystok, Waszyngtona 13, 15-269 Białystok, Poland; natalia.kurylonek@gmail.com; 3 Department of Endocrinology, Diabetology and Internal Medicine, Medical University of Bialystok, ul. M. Skłodowskiej-Curie 24A, 15-276 Białystok, Poland

**Keywords:** rheumatoid arthritis, single-nucleotide polymorphisms, interleukin-1, interleukin-6, interleukin-8, interleukin-15, interleukin-17, interleukin-18, interleukin-23, tumor necrosis factor-alpha

## Abstract

Conducted studies highlight that a mixture of genetic and environmental factors is responsible for rheumatoid arthritis (RA) development. This study aimed to analyze the available literature for the relationship between, on the one hand, single-nucleotide polymorphisms (SNPs) in the proinflammatory cytokines genes *interleukin-1* (*IL-1*), *-6, -8, -15, -17, -18*, and *-23*, and *tumor necrosis factor-alpha* (*TNF-α*), and on the other hand, RA susceptibility, severity, and patients’ response to applied treatment. The PubMed database was searched for sources. Preference was given to articles which were published within the past 20 years. Data indicate that the relationship between selected SNPs in proinflammatory cytokines genes and susceptibility to developing RA is inconclusive, and it depends on the ethnicity of the population. Although the allelic and genotypic frequencies of many SNPs in proinflammatory cytokines genes analyzed did not differ between RA patients and healthy controls, deeper analysis showed that these polymorphisms have a relationship with clinicopathological features of RA. SNPs in proinflammatory cytokines genes also “modify patients’ response” to applied treatment. Further studies, on larger cohorts of subjects and in different populations, should be conducted to elucidate the role of SNPs in *IL-1, -6, -8, -15, -17, -18*, and *-23*, and *TNF-α* genes in RA patients.

## 1. Introduction

Rheumatoid arthritis (RA) is a chronic, progressive, autoimmune inflammatory process. It affects almost 1% of the population of developed countries, which makes it a significant health and economic problem. Women get sick two times more often than men [1]. The first symptoms of RA usually appear in the fourth or fifth decade of life but can occur at any age. Clinical diagnosis of RA is based on the recognition of four out of seven characteristic signs and symptoms of the disease: morning stiffness of the joints lasting more than 1 h, inflammation that occurs in at least three joint areas, arthritis of the hands, symmetrical joint arthritis, rheumatoid nodules, detection of rheumatoid factor (RF), and anticitrullinated protein antibodies (ACPA) in the serum and rheumatoid nodules and changes found in radiographic examination [2,3].

RA is characterized by inflammation of the synovial membrane of the joints, leading to its hypertrophy and thickening, destroying cartilage and articular surfaces of the bones. The pathomechanism of RA development is the same as in the course of other autoimmune diseases—the patient’s body develops an immune response against its proteins, leading to a cascade of pro-inflammatory signaling pathways [4]. In inflammatory joints, neutrophils, T cells, B cells, macrophages, osteoclasts, and synovial fibroblasts are activated, which leads to the production of cytokines and chemokines. These molecules induce the secretion of enzymes and other factors that contribute to the destruction of cartilage and bone tissue. At the same time, as a result of over-reactivity of the immune system, patients develop inflammatory changes in many organs (including the heart, lungs, blood vessels), which in many cases leads to disability and premature death [5,6].

**Figure 1:**
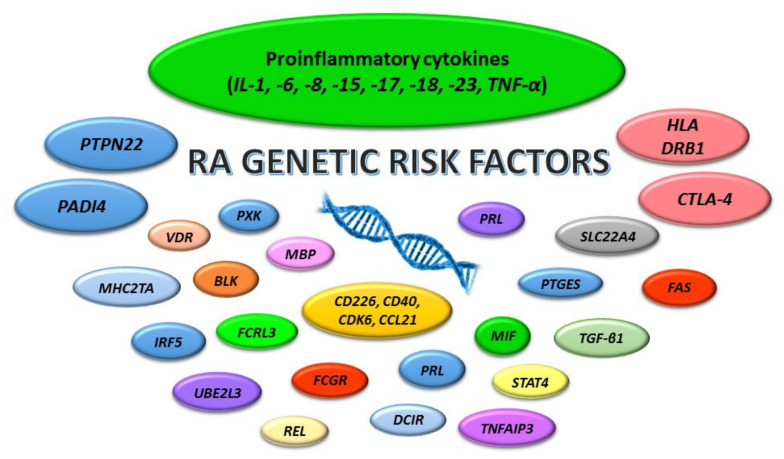
Genes associated with RA development. *BLK*—B lymphocyte kinase; *CD*—cluster of differentiation; *CTLA-4*—cytotoxic T lymphocyte-associated antigen-4; *DCIR*—dendritic cell immunoreceptor; *FAS*—FAS gene; *FCGR*—Fcγ receptor gene family; *FCRL3*—Fc receptor-like 3; *HLA*—human leukocyte antigen; *IL*—interleukin; *IRF5*—interferon regulatory factor 5; *MBP*—myelin basic protein; *MHC2TA*—major histocompatibility complex class II transactivator; *MIF*—macrophage migration inhibitory factor; *PADI4*—the type 4 peptidyl arginine deiminase; *PRL*—prolactin; *PTGES*—prostaglandin E synthase; *PTPN22*—protein-tyrosine-phosphatase, nonreceptor type 22; *PXK*—PXK domain-containing serine/threonine kinase; *RA*—rheumatoid arthritis; *REL*—v-rel reticuloendotheliosis viral oncogene homolog; *SLC22A4*—solute carrier 22 member 4 gene; *STAT4*—signal transducer and activator of transcription 4; *TGF-β1*—transforming growth factor β-1; *TNFAIP3*—tumor necrosis factor alpha-inducible protein 3; *TNF-α*—tumor necrosis factor α; *UBE2L3*—ubiquitin-conjugating enzyme *E2L 3*; *VDR*—vitamin D receptor.

The exact etiology of RA is still unknown, but a huge number of researchers highlight that a mixture of genetic and environmental factors is responsible for its development. It is indicated that the genes most associated with RA susceptibility and course are *HLA-DR, PTPTN22, CTLA-4*, and *PADI4* [4,6]. Excellent reviews regarding genetics in RA have appeared in the articles of Chatzikyriakidou et al. and Perricone et al. [7,8]. It is also indicated that among the genes predisposing a person to the development of RA are genes encoding pro-inflammatory cytokines (Figure 1) [7,9,10,11]. Therefore, our work aimed to analyze the available literature for the relationship between, on the one hand, single-nucleotide polymorphisms (SNPs) in proinflammatory cytokines genes *interleukin-1* (*IL-1*), -*6, -8, -15, -17, -18*, and *-23*, and *tumor necrosis factor-alpha* (*TNF-α*), and on the other, RA susceptibility, severity, and patients’ response to applied treatment.

### Search Strategy

We searched the PubMed database for sources using the following keywords: the pathogenesis of rheumatoid arthritis; rheumatoid arthritis and proinflammatory cytokines; proinflammatory cytokines and autoimmune diseases; the biology of IL-1, -6, -8, - 12, -15, -17, -18, -23, TNF-α; rheumatoid arthritis and IL-1, -6, -8, -12, -15, -17, -18, -23, TNF-α; rheumatoid arthritis and single nucleotide polymorphisms of *IL-1, -6, -8, -12, -15, -17, -18, -23, TNF-α*; rheumatoid arthritis and SNPs of *IL-1, -6, -8, -12, -15, -17, -18, -23, TNF-α*. Preference was given to the sources which were published within the past 20 years.

## 2. SNPs within IL-1 Family in RA

Interleukin 1 (IL-1), as the main pro-inflammatory cytokine, initiates the cascade of the entire inflammatory process. The IL-1 family includes 11 structurally related proteins responsible for regulating the immune response. This group includes, among others: IL-1α, IL-1β, IL-18, IL-33, and an interleukin 1 receptor antagonist (IL-1Ra) [12]. The gene encoding *IL-1α*, *IL-1β*, and *IL-1Ra* is located on chromosome 2q14.1. The gene encoding *IL-18* is located on chromosome 11q23.1. The gene encoding *IL-33* is located on chromosome 9p24.1. IL-1α and IL-1β are 17 kDa proteins. The synthesis of IL-1α takes place in neutrophils, lymphocytes, monocytes, macrophages, keratinocytes, endothelial cells, and glial cells. The synthesis of IL-1β takes place mainly in monocytes and macrophages. IL-1β is one of the first cytokines secreted during inflammation and can induce the production of other cytokines [12]. The synthesis of IL-18 occurs in most cells of a healthy organism under the influence of the soluble IL-18BP protein, which is an antagonist of IL-18 [13]. Normally, the concentration of IL-18BP is higher than that of IL-18, and the ratio changes during the inflammatory process [14]. The synthesis of IL-33 takes place in various tissues, including muscle tissue or the central nervous system tissues, but it is not synthesized in leukocytes. IL-Ra has an anti-inflammatory effect by inhibiting the activity of IL-1α and IL-1β [15].

Studies have shown that IL-1 is a key mediator of the processes involved in the pathomechanism of autoimmune diseases, including RA. It was found that the concentration of IL-1 in both the plasma and synovial fluid of RA patients is significantly increased compared to healthy controls [16]. According to Harrison et al. [17], polymorphisms in the IL-1 gene may influence the susceptibility of developing RA in a European population.

**Figure 2:**
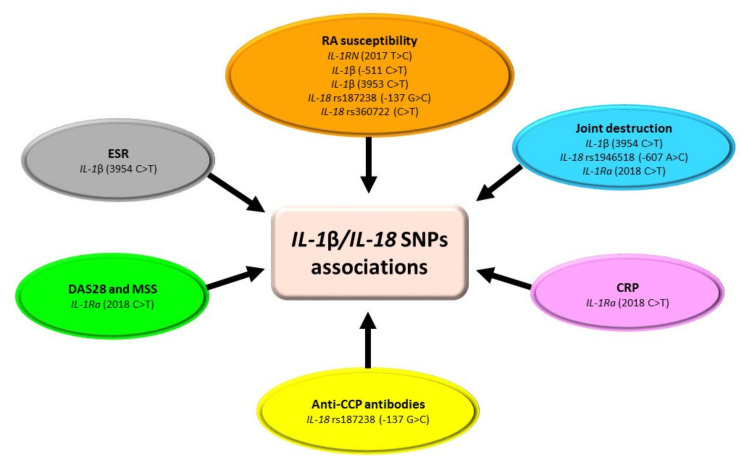
Association of *IL-1*β/*IL-18* SNPs with RA. Figure briefly summarizes information from Table 1. For precise study results and abbreviations explanation, please refer to Table 1.

The most often studied SNPs of the *IL-1* gene in RA are *IL-1*β (−511 C > T), *IL-1*β (3953 C > T), *IL-1Ra* (2018 C > T), *IL-18* rs1946518 (−607 A > C), and *IL-18* rs187238 (−137 G > C) (Table 1, Figure 2) [18,19,20,21,22,23,24,25]. However, the relationship between selected SNPs in the *IL-1* gene and susceptibility to developing RA and the severity of the disease is inconclusive, and it depends on the ethnicity of the population [17]. Only in the Turkish population was an association between the SNPs of *IL-1*β (−511 C > T) and *IL-1*β (3953 C > T) and RA shown. Arman et al. [18] indicated that the CT genotype of the *IL-1*β −511 gene variant may play a protective role for healthy people against disease development. This could be explained by fact that the heterozygous genotype of this variant can inhibit the expression of the *IL-1*β gene via the inhibition of transcription factors’ binding. The authors also showed that subjects with TT genotype of *IL-1*β 3953 polymorphism were more susceptible to developing RA. A possible explanation is that the T allele or homozygous genotype can enhance the transcription activity of the *IL-1*β gene [18]. In the remaining studied populations, Dutch, French, Italian, and Colombian, an association between *IL-1*β (−511 C > T) and/or *IL-1*β (3953 C > T) gene polymorphisms and RA susceptibility was not found [19,20,21,22]. Regarding the *IL-1Ra* (2018 C > T) SNP, it was shown that the studied polymorphic variant was not related to RA susceptibility [20,23]. However, in the Malaysian population, the CT genotype was associated with a higher RA activity based on DAS28 (28 joint-based Disease Activity Score) and joint damage based on MSS (Modified Sharp Score) [23]. It is hypothesized that the CT genotype of the *IL-1Ra* 2018 gene variant increases the production of IL-1 by monocytes [23]. Research results regarding the role of *IL-18* rs1946518 and *IL-18* rs187238 SNPs are inconsistent [11,24,25]. Unlike the Spanish population [24], in the Polish population, an association between *IL-18* rs187238 polymorphism and RA susceptibility was found [11]. Pawlik et al. [25] showed that genotype CC and diplotypes AG/AG and AC/AC at *IL-18* of rs187238 variant are significantly less common in RA patients compared to the control group. Moreover, in the Polish population, an association between *IL-18* rs187238 SNP and age at the time of RA diagnosis and the frequency of anti-CCP (anti-cyclic citrullinated peptide) antibodies in RA patients was found [25]. The same study also revealed that *IL-18* rs1946518 gene polymorphism was associated with joint damage in RA patients [25]. The results of these studies suggest that both *IL-18* rs1946518 and *IL-18* rs187238 polymorphisms may influence the course and severity of RA [25]. However, the research undoubtedly requires confirmation on a larger group of patients.

## 3. SNPs of IL-6 in RA

Interleukin 6 (IL-6) is a glycosylated polypeptide with a molecular weight of about 28 kDa, composed of 184 amino acids. The gene encoding *IL-6* is located on chromosome 7p15.3 and consists of 4 introns and 5 exons. The production of IL-6 is mainly stimulated by IL-1, TNF-α, interferons, lipopolysaccharides, and viruses [26]. IL-6 is produced by cells of the immune system—neutrophils, T and B lymphocytes, monocytes, and NK cells—but also other cells—fibroblasts, osteoblasts, keratinocytes, vascular endothelial cells, and even some neoplastic cells [27]. The receptor for IL-6 consists of two subunits: gp130, the main function of which is to transmit a signal to the interior of cells, and α-gp80, which recognizes and binds IL-6. The gp130 subunit is present on the surface of most cells, while α-gp80 only appears on neutrophils, lymphocytes, monocytes, and hepatocytes [28]. IL-6 has both pro-inflammatory and anti-inflammatory properties. It participates in acute phase reactions, and depending on the type of cells it affects, it can lead to their activation, proliferation, differentiation, and apoptosis [29].

Currently, IL-6 is considered an important marker of many disease states. IL-6 has been found to play an important role in the pathogenesis of RA by initiating neutrophil migration and osteoclast maturation, resulting in synovitis, joint destruction, and pannus formation [30]. Elevated levels of IL-6 are observed in both the synovial fluid and serum of RA patients, and the concentrations in the fluid are much higher than in the serum, which may indicate its local production [31].

**Figure 3:**
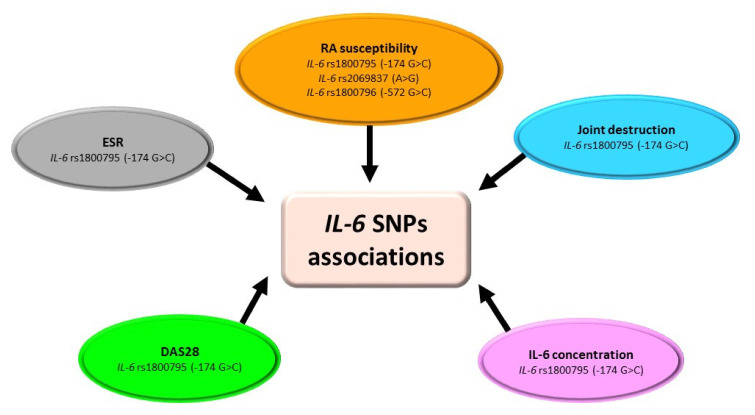
Association of *IL-6* SNPs with RA. Figure briefly summarizes information from Table 2. For precise study results and abbreviations explanation, please refer to Table 2.

The most often studied SNPs of the *IL-6* gene in RA are *IL-6* rs1800795 (-174 G > C) and rs1800796 (-572 G > C) (Table 2, Figure 3) [9,10,32,33,34,35,36,37]. Conducted studies showed that both allelic and genotypic frequencies of rs1800795 SNP did not differ between RA patients and healthy controls in Polish, Turkish, Mexican, and Indian populations [10,32,33,34,35]. Opposite results were obtained in the Chinese Han and Egyptian populations [9,36]. However, in those populations where *IL-6* rs1800795 SNP was not associated with increased susceptibility for RA risk, deeper analysis showed that this polymorphism has a relationship with clinicopathological features of RA [10,32,35]. Unfortunately, obtained data are inconsistent, as Wielińska et al. [10] indicated a favorable effect of the G allele on the RA activity, which is contrary to results obtained by Pawlik et al. [32]. Another single-nucleotide polymorphism in the *IL-6* gene widely analyzed in RA patients is *IL-6* rs1800796 SNP [9,33,34,37]. Studies showed that allelic and genotypic frequencies of *IL-6* rs1800796 polymorphism did not differ between RA patients and healthy controls in Turkish, Mexican, and Egyptian populations [9,33,34], which is contrary to the Chinese Han population [37]. Moreover, in Turkish RA patients, *IL-6* rs1800796 SNP was not associated with age, disease-onset, RF, or the presence of radiological erosions [33]. On the other hand, in the Chinese Han population, *IL-6* rs1800796 polymorphism was significantly associated with increased risk of RA among younger individuals and in males [37]. Nevertheless, the question of whether *IL-6* rs1800795 and *IL-6* rs1800796 SNPs are crucial and necessarily sufficient for RA diagnosis and progression requires further studies.

## 4. SNPs of IL-8 in RA

Interleukin 8 (IL-8/CXCL8) is a protein belonging to the chemokine family with a molecular weight of 8–9 kDa [38,39]. It consists of two polypeptides: Ser-IL-8 composed of 72 amino acids and Ala-IL-8 composed of 77 amino acids [40]. The gene encoding *IL-8* is located on chromosome 4, on the 4q13.3 region. So far, two receptors for IL-8 have been discovered, CXCR1 and CXCR2, and CXCR1 is a specific receptor for this chemokine [41]. IL-8 is one of the most potent chemotactic agents and is produced by neutrophils, monocytes, macrophages, endothelial cells, and fibroblasts. The main function of IL-8 is to activate neutrophils, which stimulates the process of chemotaxis and degranulation and enables the release of lysosomal enzymes. It is also responsible for the inhibition of neutrophil adhesion to the vascular endothelium and stimulates angiogenesis. However, to a lesser extent, IL-8 also acts on basophils, NK cells, and T cells. It causes the release of mediators of anaphylactic reaction from basophils as well as the passage of circulating T lymphocytes through the endothelium [42].

It has been found that patients with RA have an increased concentration of IL-8 both in the serum and in the synovial tissue [43]. Recent studies also show that IL-8 production in the synovial tissue of RA patients is induced by anti-CCP, leading to osteoclast activation [44].

According to our best knowledge, two sets of data have been used in evaluating the influence of the *IL-8* gene polymorphisms on the risk of RA [45,46]. Lo et al. [45] showed that in the Taiwanese population (199 RA patients vs. 130 healthy subjects), 2767 A > G polymorphism in the 3′-untranslated region (UTR) of the *IL-8* gene is not associated with the risk of RA developing. However, the authors found that patients with *IL-8* 2767 AA genotype developed RA at a younger age than patients without that genotype. Their findings suggest that in the Taiwanese population, *IL-8* 2767 AA genotype may influence the etiopathology of RA [45]. Another SNP of the *IL-8* gene analyzed in RA is *IL-8* rs2227306 (781 C > T). Emonts et al. [46] showed that in the Caucasian population (376 RA patients vs. 463 healthy subjects), *IL-8* rs2227306 gene polymorphism was related to the increased RA susceptibility, but the CC genotype of *IL-8* rs2227306 was associated with early onset of RA diagnosis compared to the CT and TT genotypes [46]. Nevertheless, due to limited literature data, further studies are needed to define the role of SNPs in the IL-8 gene in RA patients.

## 5. SNPs of IL-15 in RA

Interleukin 15 (IL-15) is a pro-inflammatory cytokine with multi-directional effects on the immune system. It is a glycoprotein with a molecular weight of 14–15 kDa, consisting of 114 amino acids. It has a structure of four α helices connected by loops. The gene encoding *IL-15* is located on chromosome 4, on the 4q31.21 region [47]. The IL-15 receptor (IL-15R) consists of three subunits: the IL-15-specific IL-15Rα subunit, the IL-15 and IL-2 subunit IL-2/IL-15Rβ, and the γ subunit, which is involved in the binding of six cytokines (IL-2, -4, -7, -9, -15, -21) [48]. IL-15 is responsible for the formation and differentiation of NK cells. It has chemotactic properties for them, increases their cytotoxic activity, and determines the secretion of IFN-γ by them. This cytokine also activates neutrophils and macrophages. In addition, it has a significant effect on T lymphocytes—it leads to their proliferation, conditions their growth, supports their activation, and is a chemotactic factor for them [49]. Physiologically, IL-15 is produced, for example, by monocytes, macrophages, dendritic cells, skeletal muscle cells, kidneys, lungs, heart, and placenta [50].

Kurowska et al. [51] showed that the combined evaluation of the concentration of IL-15, RF, and ACPA was characterized by a higher diagnostic accuracy in predicting the development of RA in patients with undifferentiated arthritis than the assessment of only autoantibodies. Therefore, IL-15 may be an additional diagnostic marker helpful in the assessment of the early stage of RA [51].

**Figure 4:**
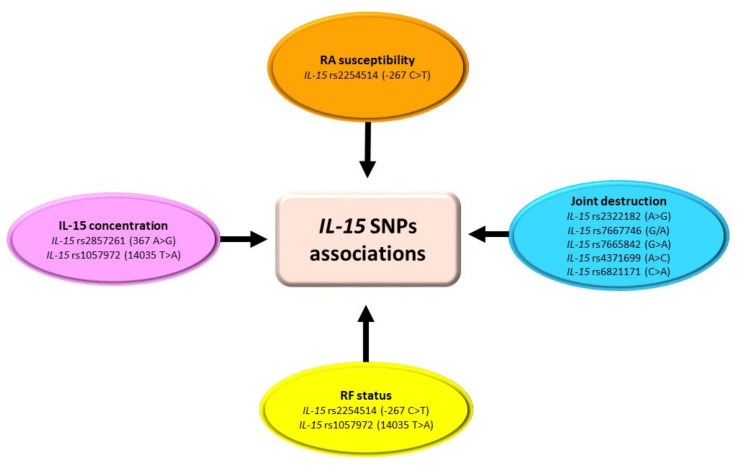
Association of *IL-15* SNPs with RA. Figure briefly summarizes information from Table 3. For precise study results and abbreviations explanation, please refer to Table 3.

In the available literature, only a few studies evaluate single-nucleotide polymorphisms of the *IL-15* gene (Table 3, Figure 4) [2,52,53,54]. Except for *IL-15* rs2254514 (−267 C > T) SNP [2], none of the tested genetic variants were predictive for RA diagnosis. Moreover, there are conflicting data regarding the relationship between *IL-15* genetic variants and the severity of joint destruction [52,53,54]. So far only Knevel et al. indicated that some of the *IL-15* gene polymorphisms were significantly associated with rate of joint destruction in RA patients [53]. An explanation of this may be fact that *IL-15* exhibits genetic heterogeneity among different ethnic populations [53,54]. On the other hand, IL-15 takes a role not only in inflammation and immune defense, but most of all in normal homeostasis. Thus the expression of IL-15 could be strictly controlled and genetic variants affecting the *IL-15* gene might be conservative [52]. Nevertheless, further studies should be conducted to unexclusively elucidate whether *IL-15* gene variants are related to increasing RA susceptibility, as well as expression, activity, and other biological functions of IL-15.

## 6. SNPs within IL-17 Family in RA

The IL-17 family consists of six cytokines (IL-17A, IL-17B, IL-17C, IL-17 D, IL-17E, IL-17F), which differ in structure and function. IL-17A is believed to be the prototype of this family, while the functions of the other cytokines are not yet well understood [55]. IL-17A is a 35 kDa glycoprotein consisting of 150 amino acids. The closest similarity to IL-17A shows IL-17F. Both of these cytokines can be secreted as homo- or heterodimers linked by disulfide bridges. The genes encoding *IL-17A* and *IL-17F* are on chromosome 6 (6p12) [56]. The sources of IL-17A are mainly Th17 lymphocytes, but also Tc lymphocytes, NK cells, eosinophils, and neutrophils [57]. The IL-17A receptor (IL-17AR) is a type 1 transmembrane protein. IL-17AR is present, among others, on leukocytes, epithelial and endothelial cells, keratinocytes, fibroblasts, and hematopoietic cells. IL-17AR can bind not only IL-17A, but also IL-17B, IL-17E, and IL-17F [58].

The role of IL-17 in the pathomechanism of RA development was first suggested by Chabaud et al. [59], who demonstrated the presence of this cytokine in the synovial fluid of RA patients. Further studies have shown that in rheumatic diseases, the locally produced IL-17 acts pathogenically by stimulating the production by synoviocytes and chondrocytes of other pro-inflammatory cytokines, inflammatory mediators (prostaglandin E2/PGE2), and proangiogenic factors (vascular endothelial growth factor/VEGF), which intensifies and supports inflammatory response. In addition, it induces the synthesis of MMPs (matrix metalloproteinases) by these cells, and increases the expression of RANKL (receptor activator for nuclear factor κB ligand), contributing to the destruction of cartilage and articular bone [60].

**Figure 5:**
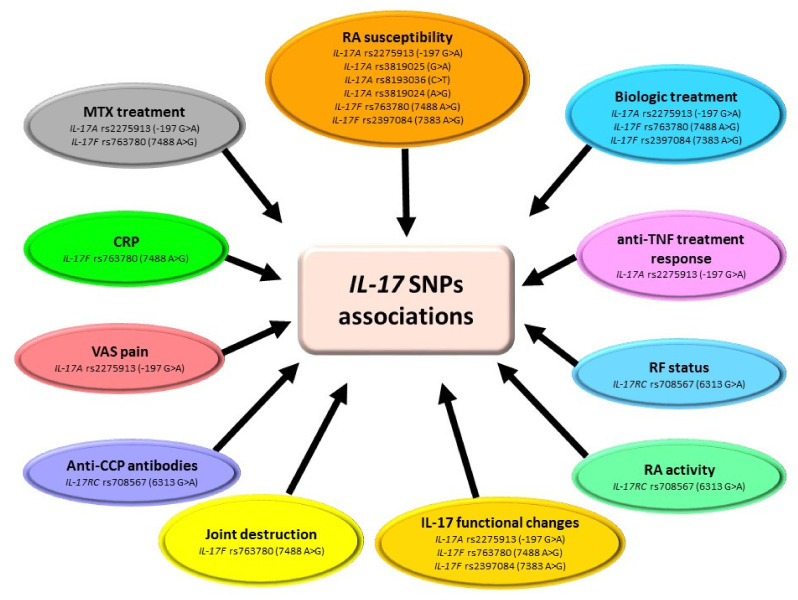
Association of *IL-17* SNPs with RA. Figure briefly summarizes information from Table 4. For precise study results and abbreviations explanation, please refer to Table 4.

Among the *IL-17* family, the most often studied SNPs were *IL-17A* rs2275913 (−197 G > A), *IL-17F* rs763780 (7488 A > G), *IL-17F* rs2397084 (7383 A > G), *IL-17A* rs4711998 (A > G), and *IL-17A* rs3819024 (A > G) (Table 4, Figure 5) [61,62,63,64,65,66,67,68,69]. Analysis conducted by researchers showed that *IL-17A* rs2275913 SNP is significantly associated with RA risk in Norwegian, New Zealander [61], Chinese [62], Brazilian [63], and Pakistani [64] populations. In the Polish population, GG genotype of the *IL-17A* rs2275913 gene variant was associated with worse patients’ responses to anti-TNF treatment [65]. On contrary, in the Tunisian population, the above-mentioned polymorphism was related to enhanced patients’ response to biological and MTX treatment [66]. *IL-17F* rs763780 SNP is significantly associated with RA risk in Polish [65], Tunisian [66], and Pakistani [64] populations. Moreover, in Tunisian RA patients, it was also associated with enhanced patients’ response to MTX treatment [66], while in Polish RA patients it was associated with an increased number of involved joints [67]. *IL-17F* rs2397084 SNP is significantly associated with RA risk in the Tunisian [66] and Pakistan [64] populations. Additionally, patients with the above-mentioned polymorphism respond better to biological treatment [66].

Presented studies confirmed that both *IL17A* and *IL17F* are potential candidate genes involved in RA susceptibility and clinical manifestation of disease. They also modify the response to RA treatments. However, larger-scale studies from more diverse ethnic populations should be performed to determine if these polymorphisms may have a link with RA pathology, course, and response to treatment.

## 7. SNPs of *IL-23R* in RA

Interleukin 23 (IL-23) is a cytokine that belongs to the IL-12 family [70]. The gene encoding *IL-23* is located on chromosome 12, on the 12q13.3 region. IL-23 is a heterodimer consisting of two subunits: p19, which is homologous to IL-12 p35, and the p40 subunit common to IL-23 and IL-12 [71]. The p19 subunit itself has no biological activity, but due to its association with the p40 subunit via a disulfide bridge, it forms a biologically active IL-23 that can bind to a receptor composed of the IL-12Rβ1 subunit and the IL-2-specific IL-23R subunit. [72]. Stimulated macrophages and dendritic cells produce IL-23, resulting in the differentiation of Th17 lymphocytes [73]. The Th17 lymphocyte subpopulation plays an important role in the inflammation process as it is involved in the production of significant amounts of IL-17A and IL-17F, which in turn enhance the stimulation of T lymphocytes, resulting in the production of other inflammatory mediators (IL-1, IL-6, TNF-α) [74].

The presence of IL-23 was detected in the serum and synovial fluid of RA patients. In addition, it has been shown that the p19 subunit of IL-23 is overexpressed in synovial fibroblasts of RA patients [75]. In vitro cultured synoviocytes from RA patients produced IL-23 and IL-23R, while blockade of IL-23 resulted in a reduction in the production of IL-1, IL-6, and TNF-α [76]. In the context of the pathomechanism of RA formation, IL-23 has been shown to stimulate the differentiation of osteoclast precursors through the production of IL-17 and other pro-inflammatory cytokines in a Th17-dependent manner. Consequently, IL-23 has become an attractive therapeutic target for inhibiting bone destruction in RA [75].

**Figure 6:**
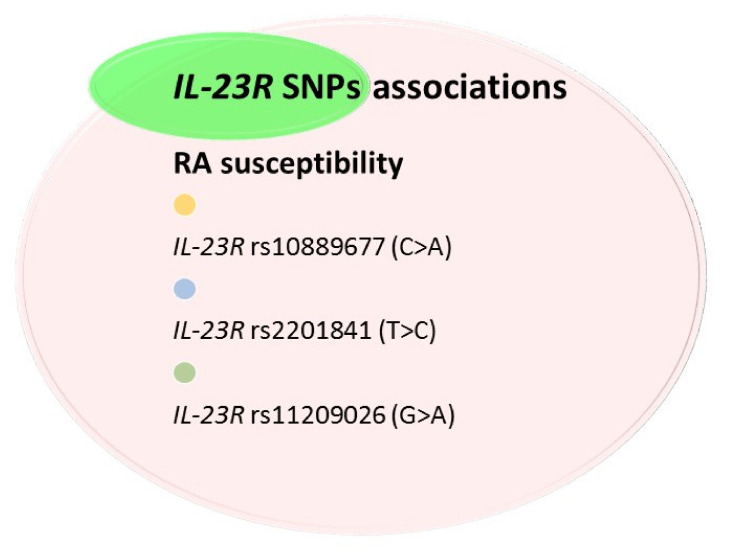
Association of *IL-23R* SNPs with RA. Figure briefly summarizes information from Table 5. For precise study results and abbreviations explanation, please refer to Table 5.

The available literature studies are mainly focused on *IL-23R* polymorphisms’ analysis (Table 5, Figure 6) [63,65,77,78,79,80,81]. Conducted research showed that in Korean [77], Spanish [78], and Polish [65] populations, *IL-23R* gene does not seem to be associated with RA predisposition. The lack of association between *IL-23R* SNPs and RA could be due to the fact that the activation of the IL-23/IL-17 pathway is important rather in early inflammatory immune response [77]. One another possible explanation is that *IL-23R* gene polymorphisms were associated with organ-specific autoimmune diseases, such as ankylosing spondylitis, psoriasis, or inflammatory bowel disease [79], but not with systemic autoimmune diseases, such as RA and systemic lupus erythematosus (SLE) [78]. Therefore, a conclusion can be drawn that IL-23, contrary to IL-12, is associated with the local inflammatory response.

Interestingly, in Hungarian [80], Egyptian [81], and Brazilian [63] populations, some SNPs of the *IL-23R* gene could be associated with RA susceptibility. For example, *IL-23R* rs11209026 (G > A) SNP is very frequent in the Egyptian population [81]. Functional studies indicated that it strongly influences the binding of IL-23 to its receptor [82]. Other *IL-23R* SNPs associated with RA predisposition are *IL-23R* rs10889677 (2199 C > A) and *IL-23R* rs2201841 (C > T) [80,81]. It was shown that the C allele and CC genotype of *IL-23R* rs2201841 were less frequent in RA patients compared to the control group, which may indicate the protective role of this allele [80,81]. Moreover, it is hypothesized that the presence of haplotypes AA of rs10889677 and CC of rs2201841 in the same subjects seems to be associated with RA predisposition [80].

## 8. SNPs of *TNF-α* in RA

Tumor necrosis factor-alpha (TNF-α) is one of the 20 proteins belonging to the TNF family. This molecule is a homotrimer composed of three identical subunits, consisting of 157 amino acid residues with a mass of 17 kDa each [83]. The gene encoding *TNF-α* is located on chromosome 6, on the 6p21.33 region [84]. TNF-α is produced as an integral type II membrane protein, which, under the influence of TACE metalloproteinase (TNF-α converting enzyme), is released into the environment in a soluble form [85]. Although the main source of TNF-α are monocytes and macrophages, its synthesis also occurs with the participation of mast cells, NK cells, T and B lymphocytes, neutrophils, fibroblasts, osteoclasts, and endothelial cells [86]. The biological activity of TNF-α is possible due to its association with two receptors: TNFRI and TNFRII [83]. Both of these receptors can be dissolved by TACE and form the so-called soluble forms (sTNFRI and sTNFRII), which are natural inhibitors of TNF-α that limit its activity [87].

TNF-α is a cytokine with pleiotropic activity. It is responsible, inter alia, for the synthesis of chemokines, prostaglandins, and other pro-inflammatory cytokines. It also takes part in the activation of lymphocytes, monocytes, and macrophages and enhances the expression of adhesion molecules [83]. TNF-α as a key mediator of the inflammatory process is involved in the development of arthritis. It was found that TNF-α stimulates the synthesis of collagenases in synovial fibroblasts and chondrocytes of the articular cartilage, and causes the activation of osteoclasts, leading to joint cartilage damage, synovial hyperplasia, bone resorption, and erosion in them [88].

**Figure 7:**
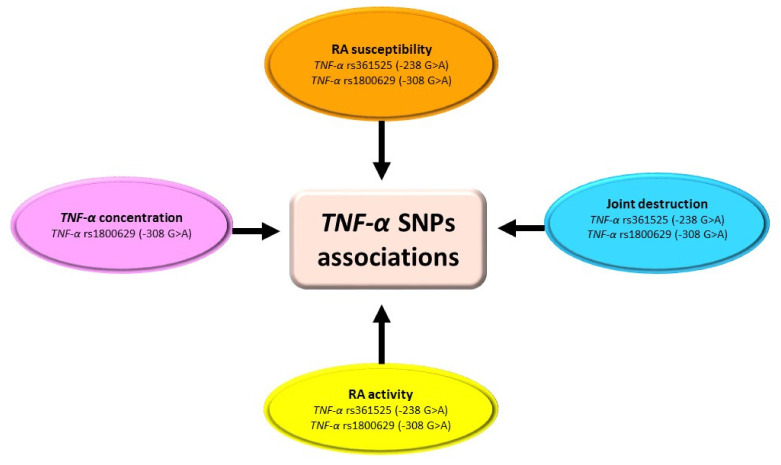
Association of *TNF-α* SNPs with RA. Figure briefly summarizes information from Table 6. For precise study results and abbreviations explanation, please refer to Table 6.

Among *TNF-α* SNPs analyzed, the most often studied were *TNF-α* rs361525 (−238 G > A), *TNF-α* rs1800629 (−308 G > A), and *TNF-α* rs1800750 (−376 G > A) SNPs (Table 6, Figure 7) [89,90,91,92,93,94]. Studies indicated that *TNF-α* rs361525, as well as *TNF-α* rs1800629 gene variations, depend on ethnicity [89,90,91]. In the Latin American, but not Turkish, population, *TNF-α* rs361525 SNP could be a genetic factor associated with increased susceptibility to RA [90,91]. In the European population, the presence of the GG genotype of *TNF-α* rs361525 SNP is a genetic factor responsible for increased joint damage in RA patients as compared to the GA genotype [89]. However, it should be noted that none of the conducted studies provide information on the influence of the *TNF-α* rs361525 variations on protein production or function. Thus, further studies should be conducted to elucidate the role of this polymorphism on the physiological role of TNF-α.

In the North Indian [92] and Egyptian [93] populations, the polymorphic variant of rs1800629 of the *TNF-α* gene could be a significant genetic risk factor associated with increased susceptibility to RA, but not in the Turkish [91], Tunisian [94], and Latin American populations [90]. In addition, data regarding the allele frequency of this polymorphism are contradictory. Gheita et al. [93] indicated that none of the controls had the A allele of *TNF-α* rs1800629 SNP. Opposite results were obtained by Gambhir et al. [92], who showed that the frequency of the A allele of *TNF-α* rs1800629 SNP was significantly lower in RA patients compared to the control group. Analyzing the possible cause of this discrepancy, it should be noted that the study population of Gheita et al. was relatively small (43 RA patients vs. 30 controls) [93]. Data regarding the role of *TNF-α* rs1800629 SNP in the RA course is also contradictory. In Latin American [90] and Tunisian [94] populations, the A allele of *TNF-α* rs1800629 SNP is associated with the susceptibility to develop more severe RA. However, these results were not confirmed in the North Indian population [92]. Thus, further studies should be conducted to exclusively elucidate the association of *TNF-α* rs1800629 SNP with clinical manifestations of RA.

## 9. Conclusions

Our work is the first to give an overview of the relationship between single-nucleotide polymorphisms in proinflammatory cytokines (IL-1, -6, -8, -15, -17, -18, -23, TNF-α) genes and RA susceptibility, severity, and patient’s response to applied treatment. Data presented in this article indicate that the association between selected SNPs in proinflammatory cytokines genes and susceptibility to developing RA is inconclusive, and it depends on the ethnicity of the population. However, the studied polymorphisms may be important factors affecting the course and severity of RA. Although the fact that allelic and genotypic frequencies of many SNPs in proinflammatory cytokines genes analyzed did not differ between RA patients and healthy controls, their deeper analysis showed that these polymorphisms have a relationship with clinicopathological features of RA, such as number of swollen and tender joints, index of joint destruction, ESR values, DAS28 score, and lasting of morning stiffness. SNPs in proinflammatory cytokines genes can also “modify patients’ response” to applied treatment.

In summary, our literature research indicated that chosen SNPs of inflammatory cytokines genes would be significant genetic factors modulating RA course. Thus, better exploration of their polymorphic variants would gain more insight into the genetic architecture of RA. However, further studies should be conducted on larger cohorts and in different populations of patients. Taking into account that epigenetic mechanism such as DNA methylation, post-transcriptional, and post-translational modifications regulating the gene expression may influence proteins production and levels, these studies should definitely be expanded with regards to functional analysis. The current review also indicated that some SNPs of inflammatory cytokines genes could be promising biomarkers to realize the so-called personalized medicine in RA patients. Identifying suitable individuals for specific treatment is very important in modern pharmacotherapy to maximize its efficacy. Therefore, the issue of the possible association of polymorphic variants of proinflammatory cytokines genes with the response and forecasting to applied treatment also requires better exploration in RA patients.

### Future Directions

Undoubtedly, a meta-analysis of the genetic association studies of proinflammatory cytokines genes would be very informative in understanding gene–RA associations [93]. Data presented in this review indicated that initial positive associations with RA susceptibility were not reproduced in other studies. The explanation of this could be the so-called type I error, indicating that original studies are false-positive, or the so-called type II error, indicating that presented results are false-negative [95].

Critical analysis of our literature research data in terms of the possibility to statistically summarize results regarding SNPs in proinflammatory cytokines genes indicated a few factors which could make our meta-analysis untrustworthy: (i) only a single study was available for SNPs in the IL-8 gene; (ii) only some SNPs for IL-1β, IL-6, IL-17, and TNF-α have been well studied; (iii) for all cytokines, the same groups of SNPs were analyzed only twice or even only once; (iv) a small number of patients was included in some studies; (v) different ethnic groups were analyzed; and (vi) studies have potential for bias (the lack of full patients’ characteristics and inclusion/exclusion criteria). Thus, future studies regarding SNPs in proinflammatory cytokines genes analysis should be designed more rigorously to exclude low-quality research with bias, which will avoid the meta-analysis also being biased.

## Figures and Tables

**Table 1:** *IL-1*β/*IL-18* single-nucleotide polymorphisms (SNPs) in rheumatoid arthritis (RA) patients.

Author	SNPs Analyzed	Nationality/ No. of Cases	Study Results
[20]	*IL-1*β (−511 C > T) *IL-1*β (3954 C > T) *IL-1Ra* (2018 C > T)	French/297 RA patients vs. 112 healthy controls	- Allelic frequencies of *IL-1*β (−511 C > T), *IL-1*β (3954 C > T), and *IL-1Ra* (2018 C > T) SNPs did not differ between RA patients and healthy controls. - The frequency of allele T for *IL-1*β (3954 C > T) polymorphism was significantly higher in DRA as compared to NDRA patients and controls. - Neither *IL-1*β nor *IL-1Ra* concentration differed in patients grouped according to the *IL-1*β (−511 C > T), *IL-1*β (3954 C > T), or *IL-1Ra* (2018 C > T) polymorphism genotypes. *IL-1Ra* plasma concentration adjusted to ESR values was significantly lower in *IL-1*β (3954 C > T) positive than negative RA patients. - Allele T of *IL-1*β (3954 C > T) polymorphism was significantly associated with higher values of ESR, index of joint destruction, clinical activity, and disability.
[19]	*IL-1α* (4845 G > T) *IL-1β* (3953 C > T) *IL-1RN* (5111 T > C) *IL-1RN* (2017 T > C)	Dutch/312 RA patients vs. 94 incident female RA patients vs. 245 healthy controls	- Allelic and genotypic frequencies of *IL-1α* (4845 G > T), *IL-1*β (3953 C > T), and *IL-1RN* (5111 T > C) SNPs did not differ between both RA patient groups and healthy controls. - Allelic and genotypic frequencies of *IL-1RN* (2017 T > C) SNP significantly differ between RA patients and healthy controls. RA patients had a significantly higher frequency of allele C compared with controls. Allele C did not correlate with RA severity in both retrospective and prospective studies.
[21]	*IL-1*β (−511 C > T) *IL-1*β (3953 C > T)	Colombian/ 172 RA patients vs. 392 healthy controls	- Allelic and genotypic frequencies of *IL-1*β (−511 C > T) and *IL-1*β (−511 C > T) SNPs did not differ between RA patients and healthy controls. - In both RA patients and the control group there was linkage disequilibrium (LD) between (−511 C > T) and (−511 C > T) of *IL-1*β gene polymorphisms.
[24]	*IL-18* rs1946518 (−607 A > C) *IL-18* rs187238 (−137 G > C)	Spanish/362 RA patients vs. 339 healthy controls	- Allelic and genotypic frequencies of both *IL-18* (−607 A > C) and (−137 G > C) SNPs did not differ between RA patients and healthy controls. - There was no association between *IL-18* (−607 A > C) and (−137 G > C) allelic or genotypic frequencies and sex, age at the onset of the disease, the presence of SE, RF status, extraarticular manifestations, or erosive disease of RA patients.
[18]	*IL-1*β (−511 C > T) *IL-1*β (3953 C > T)	Turkish/96 RA patients vs. 104 healthy controls	- Allelic frequency of *IL-1*β (−511 C > T) SNP did not differ between RA patients and healthy controls. - Genotypic frequency of *IL-1*β (−511 C > T) SNP significantly differs between RA patients and healthy controls. The CT genotype of *IL-1*β -511 was significantly lower in RA patients compared to the control group. - Allelic and genotypic frequencies of *IL-1*β (3953 C > T) SNP significantly differ between RA patients and healthy controls. The frequency of allele T for *IL-1*β (3953 C > T) polymorphism was significantly higher in RA patients compared to the control group. TT genotype of *IL-1*β (3953 C > T) SNP was significantly higher in RA patients compared to the control group.
[22]	*IL-1*β (−511 C > T) *IL-1*β (3953 C > T)	Italian/126 RA patients vs. 178 healthy controls	- There was no significant difference in genotype and allelic distribution of the *IL-1*β (−511 C > T) and *IL-1*β (3953 C > T) SNPs between RA patients and the control group.
[11]	*IL-18* rs1946518 (−607 A > C) *IL-18* rs187238 (−137 G > C)	Polish/309 RA patients vs. 305 healthy controls	- Allelic and genotypic frequencies of *IL-18* rs1946518 SNP did not differ between RA patients and healthy controls. - Genotypic frequency of *IL-18* rs187238 SNP significantly differs between RA patients and healthy controls. CC genotype of *IL-18* rs187238 polymorphism was significantly lower in RA patients compared to the control group. - RA patients had a significantly decreased number of AC/AC and AG/AG diplotypes compared to healthy controls. - There was no association between *IL-18* diplotypes and disease activity, joint erosions, extra-articular manifestations, or RF status of RA patients.
[25]	*IL-18* rs1946518 (−607 A > C) *IL-18* rs187238 (−137 G > C) *IL-18* rs360718 (T > G) *IL-18* rs360721 (C > G) *IL-18* rs360722 (C > T) *IL-18* rs549908 (A > C) *IL-18* rs5744292 (A > G)	Polish/404 RA patients vs. 148 healthy controls	- The distributions of genotypes and haplotypes between RA patients and healthy controls did not differ, except for rs360722, in which RA patients had decreased number of TT genotype carriers. - rs1946518 CC and rs187238 GG genotypes were significantly associated with lower patients’ age at the time of RA diagnosis. - rs1946518 CC and AC genotypes were significantly associated with more frequency of erosive disease in RA patients. - rs187238 GG and GC genotypes were significantly associated with increased frequency of anti-CCP antibodies in RA patients.
[23]	*IL-1Ra* (2018 C > T)	Malaysians/77 RA patients vs. 18 healthy controls	- Allelic and genotypic frequencies of IL-1Ra (2018 C > T) SNP did not differ between RA patients and healthy controls. - CT genotype was significantly associated with increased DAS28, MSS, CRP, and the number of swollen joints in RA patients.

IL—interleukin; SNPs—single-nucleotide polymorphisms; RA—rheumatoid arthritis; IL-1Ra/IL-1RN—interleukin 1 receptor antagonist; DRA—destructive arthritis; NDRA—non-destructive arthritis; ESR—erythrocyte sedimentation rate; LD—linkage disequilibrium; SE—shared epitope; RF—rheumatoid factor; anti-CCP—anti-cyclic citrullinated peptide; DAS28—28 joint-based Disease Activity Score; MSS—Modified Sharp Score; CRP—C reactive protein.

**Table 2:** *IL-6* single-nucleotide polymorphisms (SNPs) in rheumatoid arthritis (RA) patients.

Author	SNPs Analyzed	Ethnicity/ No. of Cases	Results
[32]	*IL-6* rs1800795 (−174 G > C)	Polish/98 RA patients vs. 105 healthy controls	- Allelic and genotypic frequencies of *IL-6* rs1800795 SNP did not differ between RA patients and healthy controls. - GG genotype of the *IL-6* rs1800795 gene variant was significantly associated with the active form of RA as compared to GC and CC genotypes. - Patients with GG genotype had a significantly higher number of swollen and tender joints, increased ESR values, increased DAS28 score, and longer morning stiffness compared with patients with genotypes CC and GC.
[33]	*IL-6* rs1800795 (−174 G > C) *IL-6* rs1800796 (−572 G > C) *IL-6* rs1800797 (−597 G > A)	Turkish/178 RA patients vs. 247 healthy controls	- Allelic and genotypic frequencies of *IL-6* rs1800795, *IL-6* rs1800796, and *IL-6* rs1800797 SNPs did not differ between RA patients and healthy controls. - Genotype distributions of *IL-6* rs1800795, *IL-6* rs1800796, and *IL-6* rs1800797 SNPs in RA patients were not associated with age, disease onset, RF, or presence of radiological erosions.
[34]	*IL-6* rs1800795 (−174 G > C) *IL-6* rs1800796 (−572 G > C)	Mexican/ 137 RA patients vs. 102 healthy controls	- Allelic and genotypic frequencies of *IL-6* rs1800795 and *IL-6* rs1800796 SNPs did not differ between RA patients and healthy controls.
[36]	*IL-6* rs1800795 (−174 G > C)	Chines Han/752 RA patients vs. 798 healthy controls	- Allelic and genotypic frequencies of *IL-6* rs1800795 SNP significantly differ between RA patients and healthy controls. - The CC genotype and the C allele significantly increased risk for RA after adjustment for sex, age, BMI, smoke status, and history of heavy labor work.
[35]	*IL-6* rs1800795 (−174 G > C)	Indian/150 RA patients vs. 200 healthy controls	- Allelic and genotypic frequencies of *IL-6* rs1800795 SNP did not differ between RA patients and healthy controls. - GG genotype of the *IL-6* rs1800795 gene variant was significantly associated with ESR > 20 mm/h.
[9]	*IL-6* rs1800795 (−174 G > C) *IL-6* rs1800796 (−572 G > C)	Egyptian/99 RA patients vs. 99 healthy controls	- Allelic and genotypic frequencies of *IL-6* rs1800796 SNP did not differ between RA patients and healthy controls. - Allelic and genotypic frequencies of *IL-6* rs1800795 SNP significantly differ between RA patients and healthy controls. The CC and GC genotypes were significantly associated with RA susceptibility, and the C allele significantly increased the risk for RA.
[10]	*IL-6* rs1800795 (−174 G > C)	Polish/130 RA patients vs. 112 healthy controls	- Allelic and genotypic frequencies of *IL-6* rs1800795 SNP did not differ between RA patients and healthy controls. - Patients with CC genotype had significantly higher *IL-6* concentrations before anti-TNF treatment and significantly increased DAS28 score compared to patients carrying the G allele.
[37]	*IL-6* rs1800796 (−572 G > C) *IL-6* rs2069837 (A > G) *IL-6* rs1524107 (C > T) *IL-6* rs2069840 (G > C)	Chinese Han/508 RA patients vs. 494 healthy controls	- Stratification analysis after adjustment by age revealed that *IL-6* rs2069837 and *IL-6* rs1800796 SNPs were significantly associated with increased risk of RA among younger subjects (age ≤ 54). - Stratification analysis after adjustment by sex revealed that *IL-6* rs2069837 and *IL-6* rs1800796 SNPs were significantly associated with increased risk of RA in males.

IL—interleukin; SNPs—single-nucleotide polymorphisms; RA—rheumatoid arthritis; ESR—erythrocyte sedimentation rate; DAS28—28 joint-based Disease Activity Score; RF—rheumatoid factor; BMI—body mass index; TNF—tumor necrosis factor.

**Table 3:** *IL-15* single-nucleotide polymorphisms (SNPs) in rheumatoid arthritis (RA) patients.

Author	SNPs Analyzed	Nationality/ No. of Cases	Study Results
[52]	*IL-15* rs4956403 (C > T) *IL-15* rs3806798 (T > A) *IL-15* rs7440292 (T > C) *IL-15* rs1493012 (T > C) *IL-15* rs1493013 (T > C) *IL-15* rs2254514 (−267 C > T) *IL-15* rs2857261 (367 A > G) *IL-15* rs9282741 (C > T) *IL-15* rs9282742 (T > C) *IL-15* rs1057972 (14035 T > A) *IL-15* rs9282743 (G > T) *IL-15* rs10833 (C > T) *IL-15* rs2291596 (C > T)	Spanish/645 RA patients vs. 656 healthy controls	- *IL-15* rs4956403 and rs9282741 were not polymorphic in the studied population and thus were excluded from the association study. - Allelic and genotypic frequencies of the remaining eleven *IL-15* SNPs did not differ between RA patients and the control group. - Haplotypes frequencies did not differ between RA patients and the control group. - There was no association between *IL-15* SNPs or haplotypes and sex, age at the onset of the disease, the presence of SE, RF status, extraarticular manifestations, or erosive disease.
[53]	*IL-15* rs7667746 (G/A) *IL-15* rs7665842 (G > A) *IL-15* rs2322182 (A > G) *IL-15* rs6821171 (C > A) *IL-15* rs4371699 (A > C)	Four European cohorts (Dutch, British, Swedish)/1418 RA patients	- *IL-15* SNPs rs2322182, rs7667746, rs7665842, rs4371699, and rs6821171 were significantly associated with joint destruction. Patients homozygous for the minor alleles had a higher rate of joint destruction per year as compared to the other patients.
[2]	*IL-15* rs2857261 (367 A > G) *IL-15* rs2254514 (−267 C > T) *IL-15* rs1057972 (14035 T > A)	Czechs/156 RA patients vs. 200 healthy controls	- The allele frequency only of *IL-15* rs2254514 SNP was significantly lower in RA patients compared to the control group. - Pair-wise linkage disequilibrium between *IL-15* rs2857261 G/A gene variant and *IL-15* rs1057972 A/T gene variant was found. - Higher prevalence of *IL-15* rs1057972 A/T gene variant and *IL-15* rs2254514 C/T gene variant was found in the negative RF IgA or IgG RA patients compared to positive RF IgA or IgG RA patients. - *IL-15* rs2857261 G/A and *IL-15* rs1057972 A/T genes variant were significantly associated with *IL-15* concentration. - The highest levels of total RF and Ig-specific RFs were observed in the *IL-15* rs2254514 T/T genotype.
[54]	*IL-15* rs6821171 (C > A) *IL-15* rs1521761 (T > A)	Japanese/865 RA patients	- *IL-15* SNPs were not associated with the progression of joint destruction in the cohort of RA patients.

IL—interleukin; SNPs—single-nucleotide polymorphisms; RA—rheumatoid arthritis; SE—shared epitope; RF—rheumatoid factor; IgA—immunoglobulin A; IgG—immunoglobulin

**Table 4:** *IL-17* single-nucleotide polymorphisms (SNPs) in rheumatoid arthritis (RA) patients.

Author	SNPs Analyzed	Nationality/ No. of Cases	Study Results
[61]	*IL-17A* rs4711998 (A > G) *IL-17A* rs3819024 (A > G) *IL-17A* rs2275913 (−197 G > A) *IL-17A* rs7747909 (G > A) *IL-17A* rs8193036 (C > T)	Norwegian/ 950 RA patients vs. 933 healthy controls New Zealanders/580 RA patients vs. 504 healthy controls	- In the Norwegian population, allelic and genotypic frequencies of *IL-17A* rs2275913 SNP significantly differ between RA patients and healthy controls. The GG genotype was significantly associated with the increased risk of RA. - In the New Zealand population, allelic and genotypic frequencies of all five *IL-17A* SNPs did not differ between RA patients and healthy controls. - Combined dataset of the Norwegian and New Zealand populations showed that the GG genotype of *IL-17A* rs2275913 SNP was significantly associated with the increased risk of RA. - In both Norwegian and New Zealand populations, *IL-17A* SNPs were not associated with radiographic progression, anti-CCP, or IgM-RF.
[67]	*IL-17F* rs763780 (7488 A > G) *IL-17F* rs2397084 (7383 A > G)	Polish/220 RA patients vs. 106 healthy controls	- Allelic and genotypic frequencies of two *IL-17F* SNPs did not differ between RA patients and healthy subjects. - In both RA patients and the control group, very weak linkage disequilibrium was detected between the two SNPs analyzed. - The GG and AG genotypes of *IL-17F* rs763780 SNP were significantly associated with an increased number of involved joints and creatinine concentration.
[65]	*IL-17A* rs2275913 (−197 G > A) *IL-17F* rs763780 (7488 A > G)	Polish/89 RA patients vs. 125 healthy controls	- Allelic and genotypic frequencies of *IL-17A* rs2275913 SNP did not differ between RA patients and healthy controls. - The GG wild-type genotype of the *IL-17A* rs2275913 SNP was associated with significantly higher RA activity after 3 months of anti-TNF treatment. - G allele and GG genotype of the *IL-17F* rs763780 SNP were significantly more frequent in RA patients as compared to healthy controls.
[68]	*IL-17A* rs2275913 (−197 G > A) *IL-17F* rs763780 (7488 A > G) *IL-17F* rs2397084 (7383 A > G)	Turkish/161 RA patients vs. 88 healthy controls	- Allelic and genotypic frequencies of *IL-17A* rs2275913, *IL-17F* rs763780, and *IL-17F* rs2397084 SNPs did not differ between RA patients and healthy controls. - AA genotype of the *IL-17F* rs763780 gene variant was significantly associated with increased CRP concentration in RA patients. - AG genotype of the *IL-17A* rs2275913 gene variant was significantly associated with increased VAS pain in RA patients.
[62]	*IL-17A* rs2275913 (−197 G > A) *IL-17A* rs3819024 (A > G) *IL-17A* rs3819025 (G > A) *IL-17A* rs4711998 (A > G) *IL-17A* rs8193036 (C > T) *IL-17A* rs8193037 (G > A)	Chinese/615 RA patients vs. 839 healthy controls	- From all SNPs analyzed, allelic and genotypic frequencies only of *IL-17A* rs4711998 and *IL-17A* rs8193037 polymorphisms did not differ between RA patients and healthy controls. - The AA genotype of *IL-17A* rs2275913 and the GG genotype of *IL-17A* rs3819024 were significantly associated with decreased RA risk. - The GA genotype of *IL-17A* rs3819025 and the CT genotype of *IL-17A* rs8193036 were significantly associated with increased RA risk. - Stratified analyses according to age, sex, DAS28, functional class, RF, CRP, ESR, and ACPA status showed that the A allele of *IL17A* rs3819025 gene variant was associated with a significantly increased RA risk, especially among female patients, CRP-negative patients, ACPA-positive patients, RF-positive patients, and functional class I/II patients. The T allele of the *IL17A* rs8193036 gene variant was significantly correlated with increased RA risk, especially among younger patients, CRP-positive patients, ACPA-negative patients, RF-positive patients, functional class I/II patients, and those with a DAS28 <3.20.
[63]	*IL-17A* rs2275913 (−197 G > A) *IL-17F* rs763780 (7488 A > G)	Brazilian/127 RA patients vs. 134 healthy controls	- Allelic and genotypic frequencies of *IL-17F* rs763780 polymorphism did not differ between RA patients and healthy controls. - The GG genotype of *IL-17A* rs2275913 polymorphism was significantly associated with an increased risk of RA.
[66]	*IL-17A* rs2275913 (−197 G > A) *IL-17F* rs763780 (7488 A > G) *IL-17F* rs2397084 (7383 A > G)	Tunisians/108 RA patients vs. 202 healthy controls	- Allelic and genotypic frequencies of *IL-17A* rs2275913 polymorphisms did not differ between RA patients and healthy controls. - *IL-17F* rs763780 and *IL-17F* rs2397084 SNPs were significantly associated with RA risk. - *IL-17A* rs2275913 SNP was associated with an enhanced response to biologic and MTX treatment. - *IL-17F* rs2397084 SNP was associated with an enhanced response to biological treatment. - *IL-17F* rs763780 SNP significantly decreased good response to biologic treatment, but enhanced response to MTX treatment.
[69]	*IL-17A* rs2275913 (−197 G > A) *IL-17RC* rs708567 (6313 G > A)	Tunisians/115 RA patients vs. 91 healthy controls	- Allelic and genotypic frequencies of *IL-17A* rs2275913 and *IL-17RC* rs708567 SNPs did not differ between RA patients and healthy controls. - The frequency of the G allele of the *IL-17RC* rs708567 gene variant was significantly higher in patients with active RA. - RA patients with the G allele of the *IL-17RC* rs708567 polymorphism had significantly higher anti-CCP and IgM-RF antibodies levels.
[64]	*IL-17A* rs2275913 (−197 G > A) *IL-17F* rs763780 (7488 A > G) *IL-17F* rs2397084 (7383 A > G)	Pakistans/50 RA patients vs. 50 healthy controls	- Allelic and genotypic frequencies of *IL-17F* rs2397084 and *IL-17F* rs763780 SNPs significantly differ between RA patients and healthy controls. - Genotypic frequencies of *IL-17A* rs2275913 SNP significantly differ between RA patients and healthy controls. - Amino acid alignment showed that the three polymorphic sites *IL-17A* rs2275913, *IL-17F* rs763780, and *IL-17F* rs2397084 change the sequence of encoded amino acids, which leads to functional changes in IL-17.

IL—interleukin; SNPs—single-nucleotide polymorphisms; RA—rheumatoid arthritis; anti-CCP—anti-cyclic citrullinated peptide; RF—rheumatoid factor; IgM—immunoglobulin M; TNF—tumor necrosis factor; CRP—C reactive protein; VAS—Visual Analogue Scale; DAS28—28 joint-based Disease Activity Score; ESR—erythrocyte sedimentation rate; ACPA—anticitrullinated protein antibodies; MTX—methotrexate.

**Table 5:** *IL-23R* single-nucleotide polymorphisms (SNPs) in rheumatoid arthritis (RA) patients.

Author	SNPs Analyzed	Nationality/ No. of Cases	Study Results
[78]	*IL-23R* rs1004819 (G > A) *IL-23R* rs7517847 (A > C) *IL-23R* rs10489629 (A > G) *IL-23R* rs11209026 (G > A) *IL-23R* rs1343151 (G > A) *IL-23R* rs10889677 (C > A) *IL-23R* rs11209032 (G > A) *IL-23R* rs1495965 (A > G)	Spanish/322 RA patients vs. 342 healthy controls	- Allelic and genotypic frequencies of all nine *IL-23R* SNPs analyzed did not differ between RA patients and healthy controls. - Genotypes of all *IL-23R* SNPs did not differ, when patients were stratified according to gender, age at disease onset, presence of SE, RF, rheumatic nodules, and extra-articular disease.
[80]	*IL-23R* rs10889677 (C > A) *IL-23R* rs2201841 (T > C) *IL-23R* rs1884444 (G > T)	Hungarian/ 412 RA patients vs. 220 healthy controls	- The AA genotype of *IL-23R* rs10889677 SNP was two-fold more frequent in RA patients as compared to the control group. Similar prevalence rates were noted among the RF-positive, anti-CCP-positive, and combined RF + anti-CCP-positive RA patients. - The CC genotype of *IL-23R* rs2201841 SNP was two-fold more frequent in RA patients as compared to the control group.
[77]	*IL-23R* rs1004819 (A > G) *IL-23R* rs7517847 (T > G) *IL-23R* rs10489629 (T > C) *IL-23R* rs2201841 (G > A) IL-23R rs1343151(G > A) *IL-23R* rs11209032 (G > A) *IL-23R* rs1495965 (T > C)	Korean/1204 RA patients vs. 979 healthy controls	- Allelic and genotypic frequencies of all seven *IL-23R* SNPs analyzed did not differ between RA patients and healthy controls.
[65]	*IL-23R* rs11209026 (G > A)	Polish/89 RA patients vs. 125 healthy controls	- Allelic and genotypic frequencies of *IL-23R* rs11209026 SNP did not differ between RA patients and the control group.
[81]	*IL-23R* rs11209026 (G > A) IL-23R rs2201841(A > G) *IL-23R* rs10889677 (2199 C > A)	Egyptian/120 RA patients vs. 120 healthy controls	- The AA genotype of *IL-23R* rs11209026 SNP was more frequent in RA patients as compared to the control group.
[63]	*IL-23R* rs10889677 (2199 C > A)	Brazilian/127 RA patient vs. 134 healthy controls	- Allelic and genotypic frequencies of *IL-23R* rs10889677 SNP significantly differ between RA patients and healthy subjects. - C allele and CC genotype for the *IL-23R* rs10889677 gene variant were associated with a significantly lower risk of RA development.

IL—interleukin; SNPs—single-nucleotide polymorphisms; RA—rheumatoid arthritis; IL-23R—IL-23 receptor; SE - shared epitope; RF—rheumatoid factor; anti-CCP—anti-cyclic citrullinated peptide.

**Table 6:** *TNF-α* single-nucleotide polymorphisms (SNPs) in rheumatoid arthritis (RA) patients.

Author	SNPs Analyzed	Nationality/ No. of Cases	Study Results
[89]	*TNF-α* rs361525 (−238 G > A) *TNF-α* rs1800750 (−376 G > A)	Dutch Caucasians/ 101 RA patients vs. 403 healthy controls	- GG genotype of *TNF-α* rs361525 SNP was significantly associated with an increased rate of joint damage compared to the GA genotype, independently of HLA-DR4. - The A alleles of *TNF-α* rs361525 and *TNF-α* rs1800750 are in strong positive linkage disequilibrium. - There is no significant difference in transcriptional activity between the *TNF-α* rs361525 G allele, *TNF-α* rs361525 A allele, and *TNF-α* rs361525 A allele/rs1800750 A allele promoter/enhancers in U937, Mono Mac 6, Jurkat T, and Raji B cells.
[90]	*TNF-α* rs361525 (−238 G > A) *TNF-α* rs1800629 (−308 G > A)	Mexicans/137 RA patients vs. 169 healthy controls	- The frequency of the *TNF-α* rs361525 GG genotype was significantly higher, while the rs361525 AG genotype was significantly lower in RA patients compared to the control group. - Separate analysis within the RA group (severe/non-severe patients) showed an increased frequency of *TNF-α* rs361525 GG genotype only in non-severe RA patients compared to the control group. Decreased frequency of *TNF-α* rs361525 AG genotype was observed in both severe and non-severe RA patients compared to the control group. - Allelic and genotypic frequencies of *TNF-α* rs1800629 SNP did not differ between RA patients and the control group. - Separate analysis within the RA group (severe/non-severe patients) showed a decreased frequency of *TNF-α* rs1800629 A allele severe RA patients compared to non-severe patients. *TNF-α* rs1800629 G allele was significantly increased when compared to non-severe patients and the control group.
[91]	*TNF-α* rs361525 (−238 G > A) *TNF-α* rs1800629 (−308 G > A) *TNF-α* rs1800750 (−376 G > A)	Turkish/98 RA patients vs. 122 healthy controls	- Allelic and genotypic frequencies of *TNF-α* rs361525, *TNF-α* rs1800629, and *TNF-α* rs1800750 SNPs did not differ between RA patients and the control group.
[92]	*TNF-α* rs1800629 (−308 G > A) *TNF-α* rs1800630 (−863 C > A)	North Indians/222 RA patients vs. 208 controls	- The frequency of the A allele of *TNF-α* rs1800629 SNP was significantly lower in RA patients compared to the control group. - There was no association between *TNF-α* rs1800629 and *TNF-α* rs1800630 SNPs allelic and genotypic frequencies and bone erosions, deformities, presence of extra-articular features, or RF status.
[93]	*TNF-α* rs1800629 (−308 G > A)	Egyptians/43 RA patients vs. 30 controls	- Allelic and genotypic frequencies of *TNF-α* rs1800629 SNP significantly differ between RA patients and the control group (none of the controls had the A allele). - *TNF-α* concentration was significantly higher in RA patients with the AA genotype of *TNF-α* rs1800629 compared to those with GG and GA genotypes. - Age at disease onset was higher in RA patients with the GG genotype of *TNF-α* rs1800629 compared to those with GA and AA genotypes.
[94]	*TNF-α* rs1800629 (−308 G > A)	Tunisians/104 RA patients vs. 150 healthy controls	- Allelic and genotypic frequencies of *TNF-α* rs1800629 SNP did not differ between RA patients and the control group. - The frequencies of the A allele and AA genotype of *TNF-α* rs1800629 were significantly higher in patients with an erosive form of RA compared to the control group. - The frequencies of the G allele and GG of the *TNF-α* rs1800629 genotype were significantly lower in patients with an erosive form of RA compared to the control group.

TNF-α—tumor necrosis factor-alpha; SNPs—single-nucleotide polymorphisms; HLA-DR—human leukocyte antigen—DR isotype; RA—rheumatoid arthritis; RF—rheumatoid factor.

## Data Availability

Not applicable.
